# Gene Therapy with MiRNA-Mediated Targeting of *Mcl-1 *Promotes the Sensitivity of Non-Small Cell Lung Cancer Cells to Treatment with ABT-737

**DOI:** 10.31557/APJCP.2020.21.3.675

**Published:** 2020-03

**Authors:** Mahshid Shahverdi, Razieh Amini, Jamal Amri, Hadi Karami

**Affiliations:** 1 *Molecular and Medicine Research Center, *; 2 *Department of Molecular Medicine and Biotechnology, Faculty of Medicine, *; 3 *Traditional and Complementary Medicine Research Center, Arak University of Medical Sciences, Arak, Iran. *

**Keywords:** ABT-737, Bcl-2, Lung cancer, Mcl-1, MiRNA-101

## Abstract

**Background:**

Despite the dramatic efficacy of ABT-737, a large percentage of cancer cells ultimately become resistance to this drug. Evidences show that over-expression of *Mcl-1* is linked to ABT-737 resistance in NSCLC cells. The aim of this study was to investigate the effect of miRNA-101 on *Mcl-1 *expression and sensitivity of the A549 NSCLC cells to ABT-737.

**Methods:**

After miRNA-101 transfection, the *Mcl-1 mRNA* expression levels were quantified by RT-qPCR. Trypan blue staining was used to explore the effect of miRNA-101 on cell growth. The cytotoxic effects of miRNA-101 and ABT-737, alone and in combination, were measured using MTT assay. The effect of drugs combination was determined using the method of Chou-Talalay. Cell death was assessed using cell death detection ELISA assay kit. Results: Results showed that miRNA-101 markedly suppressed the expression of* Mcl-1 mRNA* in a time dependent manner, which led to A549 cell proliferation inhibition and enhancement of apoptosis (p < 0.05, relative to blank control). Pretreatment with miRNA-101 synergistically decreased the cell survival rate and lowered the IC_50 _value of ABT-737. Furthermore, miRNA-101 dramatically enhanced the apoptotic effect of ABT-737. Negative control miRNA had no remarkable effect on cellular parameters. Conclusions: Our findings propose that suppression of *Mcl-1* by miRNA-101 can effectively inhibit the cell growth and sensitize A549 cells to ABT-737. Therefore, miRNA-101 can be considered as a potential therapeutic target in patients with non-small cell lung cancer.

## Introduction

Lung cancer, as one of the most cause of cancer-related mortality in the world, can be divided into two different groups, small cell lung cancers (SCLC) and non-small cell lung cancers (NSCLC) (Lee et al., 2018; Huang et al., 2019). Despite significant advances, patients with NSCLC render resistance to cancer treatments (Gong et al., 2018; Zhou et al., 2019). Thus, investigation of the molecular mechanisms of tumor resistance is essential for development of novel treatment strategies.

Apoptosis or programmed cell death is a biological process involved in the normal physiological functions and tissue homeostasis. Deregulation of apoptotic mechanisms lead to the development of tumor and resistance to chemotherapy (Binju et al., 2019; Gilmore and King, 2019). Apoptosis is controlled by the complex interaction between pro-apoptotic and anti-apoptotic B-cell lymphoma-2 (Bcl-2) family proteins. Anti-apoptotic proteins including Bcl-2 itself, Mcl-1 (myeloid cell leukemia-1), Bcl-w, Bcl-xL, and Bfl-1/A1 promote cell survival, whereas pro-apoptotic proteins (e.g., Bid, Bad, Bik, Bim, NOXA, and PUMA) mediate apoptosis (Gautrey and Tyson-Capper, 2012; Jan and Chaudhry, 2019; Lim et al., 2019). Over-expression of anti-apoptotic proteins (e.g., Bcl-2, Mcl-1 or Bcl-xL) is observed in many human malignancies including NSCLC, and associated with tumor progression, poor prognosis and chemotherapy resistance (Losert et al., 2007; Akgul, 2009; Othman and Nagoor, 2014; Paul and Jones, 2014). These findings have led to the development of new anticancer strategies, including small molecule inhibitors, antisense oligonucleotides (ASOs) and small interfering RNAs (siRNAs) (Quinn et al., 2011; Rubenstein et al., 2015; Mongre et al., 2019; Naghizadeh et al., 2019).

ABT-737 is a potent and specific inhibitor of Bcl-xL, Bcl-2 and Bcl-w, which has shown single-agent activity in several cancer types including lung cancer (Avsar Abdik et al., 2019; Shen et al., 2019; Wang and Hao, 2019). However, multiple studies have documented that over-expression of *Mcl-1* confers resistance to ABT-737. Concordantly, down-regulation of *Mcl-1* by pharmacologic or genetic strategies induces sensitivity of malignant cells to the compound. Therefore, the combination of *Mcl-1* targeting and ABT-737 appears to be an efficient means of triggering apoptosis in various tumor types (Dai and Grant, 2007; Quinn et al., 2011).

MicroRNAs (miRNAs) are a family of non-coding RNAs with 18-25 nucleotides long, which bind to the 3’-untranslation regions (3’-UTR) of target transcripts to regulate gene expression, either via mRNAs degradation or translational inhibition (Hu et al., 2018; Rezaei et al., 2019; Alamdari-Palangi et al., 2020). It has been reported that miRNAs participate in a biological and pathological processes, such as cell differentiation, cell proliferation, cell growth and cell death. Aberrations in particular miRNAs expression are a hallmark of various cancer cells (Wang et al., 2014; Amri et al., 2019b). For example, miRNA-143 expression is down-regulated in NSCLC, causing elevated *c-Myc* expression, increased tumor cell growth, migration and metastasis. In contrast, over-expression of *miRNA-21* suppresses Bcl-2, inhibits apoptosis, enhances metastasis and confers multidrug resistances (Ricciuti et al., 2014; Zhang et al., 2014; MacDonagh et al., 2015; Amri et al., 2019a). In lung cancer, miRNAs are emerging as potential markers for chemoresistance and prognostic.

MiRNA-101, a tumor-suppressive miRNA, is under-expressed in various types of tumor tissues and cell lines, including lung cancer, and displays an inhibitory effect on cell apoptosis, migration, proliferation and invasion (Luo et al., 2012; Zheng et al., 2015). Moreover, it has been shown that up-regulation of miRNA-101 inhibited tumor progression, at least in part, by targeting *Mcl-1*, and associated with poorer prognosis (Su et al., 2009; Wang et al., 2010; Chen et al., 2011; Luo et al., 2012). However, the biological role of miRNA-101 on drug resistance of NSCLC cells has not yet been fully elucidated. Therefore, in the present study, the effect of miRNA-101 on sensitivity of the NSCLC cells to ABT-737 was investigated. Our data demonstrated that ectopic expression of *miRNA-101* was associated with suppression of *Mcl-1 mRNA* in tumor cells. We also found that elevated level of miRNA-101 inhibited the cell growth and enhanced the apoptotic effect of ABT-737, which suggests that miRNA-101 may play important roles in NSCLC resistance.

## Materials and Methods

*Cell culture*

Human NSCLC cell line A549 was obtained from Pasteur Institute (Tehran, Iran). The cells were maintained in RPMI-1640 medium (Sigma-Aldrich, St. Louis, MO, USA) containing 10% fetal bovine serum (FBS; Sigma- Aldrich) at 37°C in a 5% CO_2_ humidified atmosphere, with the medium changed every four days. Cells were sub-cultured at 90% confluence, seeded at 40% confluence and used in the logarithmic phase.

*Cell transfection*

The miRNA-101 mimic and negative control (NC) miRNA were purchased from Dharmacon (Lafayette, CO, USA) and transfected into cells with a final concentration of 50 nM. The sense strand sequences of miRNA-101 mimics and NC miRNA were 5’-UACAGUACUGUGAUAACUGAA-3’ and 5’-UUCUUCGAACGUGUCACGUTT-3’, respectively. All of the cell transfections were performed with Lipofectamine™2000 reagent (Invitrogen, Carlsbad, CA, USA), according to the manufacturer’s instructions. After 24 and 48 h transfection, down-regulation of Mcl-1 was assessed by quantitative real time PCR (RT-qPCR).

*RT-qPCR*

At different time points after transfection, total RNA was isolated by using YTzol reagent (Yekta Tajhiz, Tehran, Iran) and according to the manufacturer’s protocol. Complementary DNA (cDNA) was synthesized from 1 µg of purified total RNA using MMLV reverse transcriptase and oligo-dT primer (Promega, Madison, WI, USA) following the manufacturer’s instructions. The primer sequences were as follows: forward, 5’-TCCCTGGAGAAGAGCTACG-3’, reverse, 5’-GTAGTTTCGTGGATGCCACA-3’, for β-actin and forward, 5’-TAAGGACAAAACGGGACTGG-3’, and reverse, 5’-ACCAGCTCCTACTCCAGCAA-3’, for *Mcl-1*. Each real-time PCR reaction was: 1 µl of cDNA template, 0.2 µM of each primer, 12 µl of SYBR green reagent (Takara Bio, Otsu, Shiga, Japan) and 6 µl of nuclease-free distilled water. The PCR reactions were performed in the LightCycler 96 System (Roche Diagnostics GmbH, Mannhein, Germany) and the protocol parameters were as follows: initial incubation at 95°C for 5 min followed by 40 cycles of denaturation at 95°C for 5 s and annealing at 59°C for 30 s. The relative gene expression level was determined according to the 2 ^-(∆∆Ct)^ method, and the fold change in expression of Mcl-1 was normalized to an endogenous internal control (β-actin) (Livak and Schmittgen, 2001; Pirayesh Islamian et al., 2016).

*Cell growth assay*

The effect of miRNA-101 on cell growth was determined by trypan blue dye exclusion method. The cells were treated in 6-well plates as mentioned above in the experimental group for 24-120 h. Then, the cells were harvested and diluted with 0.4% trypan blue solution (Sigma- Aldrich) which was then counted under a microscope using a hemocytometer. The percent of viable cells was calculated as follows: Percent viable cells = (No of viable cells Test) / (No of viable cells Control) ×100.

*Cytotoxicity assay*

The effect of miRNA-101 on the response of lung cancer cells to ABT-737 was determined using 3-(4, 5-Dimethylthiazol-2-yl)-2, 5-Diphenyltetrazolium Bromide (MTT) assay. The experiment was subdivided into eight groups: ABT-737, NC miRNA, miRNA-101, NC miRNA and ABT-737, miRNA-101 and ABT-737, ABT-737 blank control, miRNA blank control and combination blank control. Treatment with only lipofectamine without miRNA was considered as a miRNA blank control. Treatment with 1% DMSO was served as ABT-737 blank control. Also treatment with combination of lipofectamine and DMSO was considered as a combination blank control. Briefly, cells were plated at a density of 4 × 10^3^ cells per well in 96-well plates and transfected with 50 nM of either miRNA-101 or NC miRNA. After 6 h of incubation, ABT-737 was added to the wells at a final concentration of 0, 0.25, 0.5, 1, 2, 4, 8 and 16 µM, and continued to incubate for another 24 and 48 h. Then, 10 µL of MTT solution (Sigma-Aldrich) (5 mg/ml) was added to each well and the plates were incubated for another 4 h at 37°C. Next, supernatant was discarded and 150 µL of DMSO was added to each well followed by reading the absorbance (A) at 490 nm in a microplate reader (Awareness Technology, Palm City, FL, USA). The survival rate (SR) was determined with the following formula: SR (%) = (A Experiment /A Control) × 100%. Half maximal inhibitory concentration (IC_50_) was calculated using Prism 6.01 software (GraphPad Software Inc., San Diego, CA, USA). 

*Synergy determination*

To further evaluate the effect of combining the therapies, the combination index (CI) method of Chou and Talalay was performed (Chou and Talalay, 1984). The cell survival rates were converted to Fraction affected (Fa) and analyzed using CompuSyn version 1.0 software (ComboSyn Inc., Paramus, NJ, USA). CI values less than 1, equal to 1, or greater than 1 represent synergy, additivity or antagonism, respectively.

*Apoptosis* assay

The A549 lung cancer cells (1 × 10^5^ cells/well) were placed in 12-well culture plates and then treated with miRNA-101, NC miRNA, the IC_50_ dose of ABT-737 and their combinations as described previously. Following 24 and 48 h of incubation, the cells were harvested and apoptosis was detected with the Cell Death Detection ELISA kit (Roche Diagnostics GmbH) according to the manufacturer’s protocol. This assay measures the amount of mono- and oligonucleosomes in the cytoplasm of apoptotic cells. Briefly, the cells were lysed and cell suspensions centrifuged at 200 g for 10 min. Then, 20 μL of the supernatants and 80 μL of a mixture containing anti-histone-biotin and anti-DNA-peroxidase were added to each well of streptavidin-coated plate. After incubation for 2 h in 25°C, the wells were washed and 100 μL of 2, 2-azino-bis (3-ethylbenzthiazoline-6-sulfonic acid) solution was added to each well. The reactions were stopped and absorbances were measured by using an ELISA plate reader at 405 nm. 

*Statistical analysis*

All data in this study were analyzed using GraphPad Prism software. Quantitative data were presented as mean ± standard deviation (SD). Statistical analysis was evaluated by analysis of variance (ANOVA) followed by Bonferroni’s test. Value of p less than or equal to 0.05 was considered significant.

**Figure 1:**
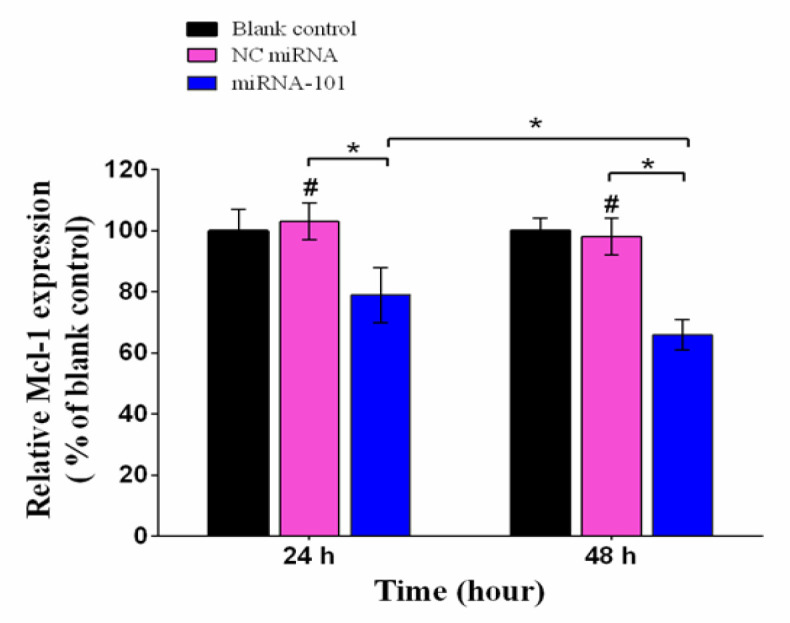
RT-qPCR Analyses of Mcl-1 mRNA in A549 Cells. To measure the expression of Mcl-1 mRNA in lung cancer cells, the A549 cells were transfected with miRNA-101 and negative control (NC) miRNA for 24 and 48 h. Relative Mcl-1 mRNA expression levels were measured by RT-qPCR using 2 ^- (∆∆Ct)^ method and β-actin as an endogenous control. Data are presented as mean ± SD (n=3). *#p > 0.05* relative to blank control; *p < 0.05

**Figure 2:**
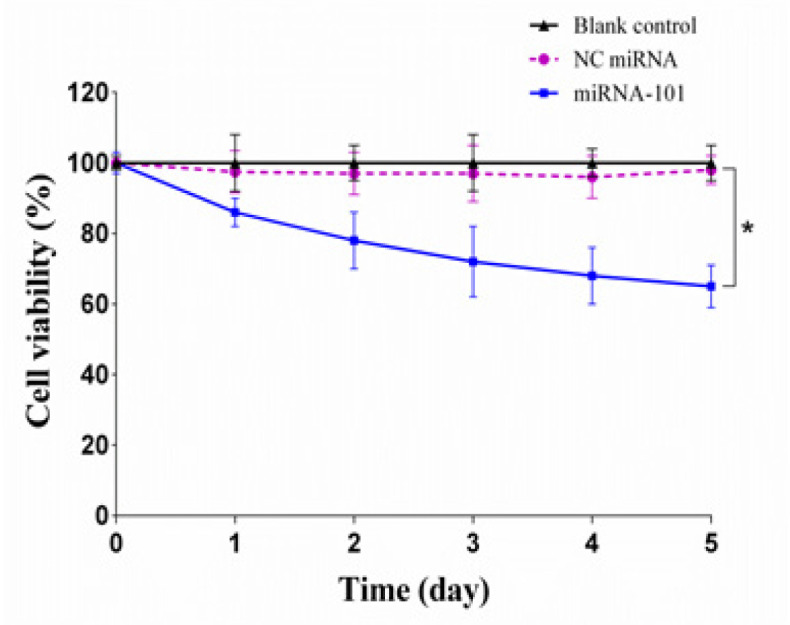
Growth Curve of A549 Cells Transfected with miRNA-101. The cells were transfected with miRNA-101 and negative control (NC) miRNA for 5 days. The cell growth rate was then determined by trypan blue assay at the end of each day. The results are represented as mean ± SD (n=3). *p < 0.05 relative to blank control or NC miRNA

**Figure 3:**
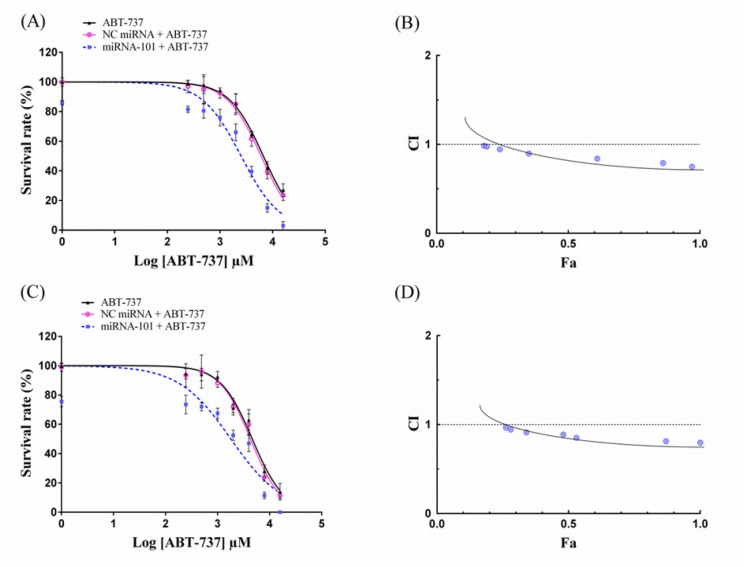
Effect of miRNA-101 in Combination with ABT-737 on Cell Survival. The A549 cells were transfected with miRNA-101 for 6 h and then treated with ABT-737 at indicated concentrations. Twenty-four (A and B) and forty-eight (C and D) hours after transfection, the cell survival was measured by the MTT assay. The cell survival curves were plotted using GraphPad 6.1 software. Data are expressed as mean ± SD of three independent experiments. The combination index (CI) was calculated using the fractional affected (Fa) values obtained from the MTT assay and CalcuSyn software

**Table 1:** IC_50_ Values of the ABT-737 Alone and in Combination with miRNAs after 24 and 48 h of Treatment of A549 Cells

Treatment	IC_50_ (µM)	
	24 h	48 h
ABT-737	6.66 ± 1.37	4.64 ± 2.90
NC miRNA and ABT-737	6.03 ± 1.80#	4.25 ± 1.78^#^
miRNA-101 and ABT-737	2.51 ± 0.80*	1.79 ± 1.23*

IC_50_ were calculated by sigmoidal dose-response (variable slope) model using GraphPad software. Data are expressed as the mean ± SD of three independent experiments. *p < 0.05 versus ABT-737; *#p > 0.05* relative to the ABT-737.

**Table 2:** CI Analysis of miRNA-101 and ABT-737 in A549 Cells

ABT-737 concentration (µM)	24 h			48 h		
	Fa	CI	Combined effect	Fa	CI	Combined effect
0.25	0.18	0.98	S	0.26	0.96	S
0.5	0.19	0.97	S	0.28	0.94	S
1	0.24	0.94	S	0.34	0.91	S
2	0.35	0.86	S	0.48	0.88	S
4	0.61	0.83	S	0.53	0.85	S
8	0.86	0.78	S	0.87	0.81	S
16	0.97	0.74	S	1	0.79	S

The combination index (CI) method of Chou and Talalay was performed using CompuSyn software. CI values less than 1, equal to 1, or greater than 1 represent synergy (S), additivity or antagonism, respectively.

**Figure 4:**
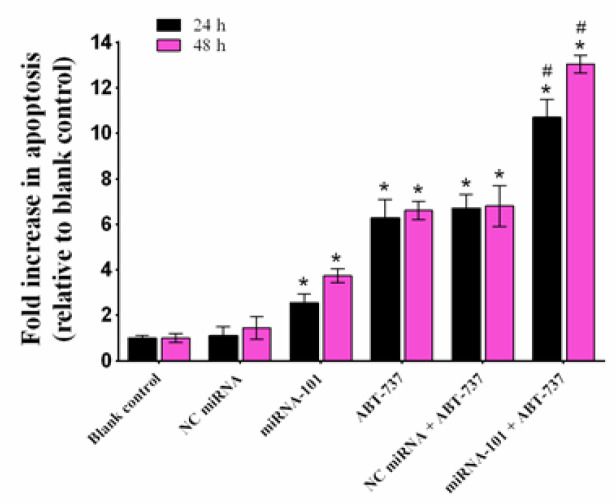
The Effect of miRNA-101 on Sensitivity of the A549 Cells to ABT-737 Mediated Apoptosis. The A549 cells were treated with negative control (NC) miRNA (50 nM), miRNA-101 (50 nM) and ABT-737 (IC50 doses of 24 and 48 h), alone and in combination. Twenty-four and forty-eight hours after transfection, apoptosis was assessed by cell death ELISA assay. The results are presented as mean ± SD (n=3). *p < 0.05 relative to blank control; *#p < 0.05* relative to miRNA-101 or ABT-737 alone

## Results

*MiRNA-101 cause down-regulation of Mcl-1 mRNA*

To analyze the effect of miRNA-101 on *Mcl-1* gene expression, A549 lung tumor cells were transfected for 24 and 48 hours with 50 nM miRNA-101 and NC miRNA. Subsequently, RT-qPCR was performed to measure expression of *Mcl-1 mRNA*. As shown in Figure 1, after treatment with 50 nM miRNA-101, the expression of *Mcl-1 *suppressed dramatically in a time-dependent way (p < 0.05; relative to the NC miRNA or blank control groups). The relative expressions of *Mcl-1 mRNA* in cells were 79.32% and 66.14% after 24 and 48 h, respectively (p < 0.05). As expected, NC miRNA had no effect on the expression of *Mcl-1 mRNA* (p > 0.05).

*MiRNA-101 showed a growth inhibitory effect in NSCLC cells*

As over-expression of *Mcl-1* is associated with the growth of NSCLC cells; we therefore investigated whether miRNA-101 could inhibit the growth of A549 cells. The cells were treated with miRNA-101 and NC miRNA and cell viability was then determined by trypan blue dye exclusion assay over a period of 5 days. The cell growth curve demonstrated that compared with the blank control group, the growth of the miRNA-101 transfected cells was inhibited notably in a time-dependent manner (p < 0.05; Figure 2). At 24 h posttransfection, the cell growth decreased to 86.40% and then to a further 65.39% at the end of the experiment (day 5). However, there was no obvious alteration in cell growth between NC miRNA transfected cells and blank control group (p > 0.05; Figure 2). 

*Enhanced sensitivity to ABT-737 by miRNA-101 in A549 cells*

We addressed whether miRNA-101 would enhance sensitivity to ABT-737 in A549 cells. The results of MTT assay showed that ABT-737 has cytotoxic effects in a dose-dependent manner (Figure 3A and 3C). Moreover 24 and 48 h exposure of the cells with miRNA-101 significantly decreased the survival of the cells (85.19% and 73.51%, respectively) relative to the blank control cells (p < 0.05). Surprisingly, the cells exposed to *miRNA-101* in the presence of ABT-737 showed a significant decrease in survival (compared with miRNA-101 or ABT-737 treated cells), and the IC_50_ values of ABT-737 were markedly decreased relative to the cells treated with ABT-737 alone (p < 0.05, Table 1). Treatment with NC miRNA had no significant effect on cell survival as well as the IC_50_ values of ABT-737.

*The combination effect of miRNA-101 and ABT-737 on lung cancer cells was synergistic*

To evaluate whether the combination of miRNA-101 and ABT-737 is synergistic, the combination index analysis was performed according to the non-constant method of Chou-Talalay. Results indicated that the combination effects of miRNA-101 (50 nM) and ABT-737 (0.25-16 µM) were synergistic with the CI values of less than 1 at any given concentration of ABT-737 (Figure 3B and 3D). CI–Fa plots further revealed that the most obvious synergism effects of 24 h (CI=0.74) and 48 h (CI=0.79) of treatments were observed at 16 µM of ABT-737 with Fa levels of 0.97 and 1, respectively (Table 2).

*MiRNA-101 sensitizes A549 cells to apoptosis induced by ABT-737*

To confirm whether the observed sensitizing effect of miRNA-101 was associated to the enhancement of apoptosis, the effects of ABT-737 and miRNA-101, alone and in combination, on apoptosis were examined using cell death ELISA assay. The results demonstrate that 24 h exposure of the cells with miRNA-101 or ABT-737 enhances apoptosis by 2.55 and 6.28 fold, respectively, compared to the blank control (p < 0.05; Figure 4). Moreover, combination of miRNA-101 and ABT-737 further increases the degree of apoptosis to 10.70 fold (p < 0.05, compared with either ABT-737 alone or miRNA-101 alone). Forty-eight hours treatment of the cells with miRNA-101 or ABT-737 alone, led to enhancement of apoptosis by 3.73 and 6.61 fold, respectively, compared to blank control (p < 0.05). Also, the combination of two agents significantly increased the extent of apoptosis relative to the monotherapy in this period of time. However, NC miRNA alone or in combination with ABT-737 had no distinct effect on apoptosis compared with the blank control or ABT-737 treated cells, respectively (p > 0.05; Figure 4). Therefore, these findings demonstrate that the sensitization effect of miRNA-101 in A549 cells is linked to the augmentation of apoptosis.

## Discussion

Non small cell lung cancer (NSCLC), which accounts for approximately 80% of all lung cancer cases, is the number one cause of cancer deaths worldwide for both men and women (Zhang et al., 2014; MacDonagh et al., 2015). Despite an initial response of the tumor to therapy, response rates were often low, due to the development of resistance (Yu and He, 2013). The precise mechanisms underlying NSCLC resistance remain unclear.

ABT-737 is one of the well studied BH3 mimetics that binds with strong affinity to Bcl-2 family anti-apoptotic proteins Bcl-2, Bcl-xL, and Bcl-w, but not to Mcl-1. ABT-737 was effective in inducing cytotoxicity as a single agent in vitro and in vivo in several types of cancer such as glioblastoma, leukemia and lymphoma, multiple myeloma and SCLC (Su et al., 2009; Quinn et al., 2011). However, a large percentage of cancers are resistant to ABT-737. There are clear evidences that show over-expression of *Mcl-1* confers resistance to ABT-737. Concordantly, adding drugs that down-regulate Mcl-1 sensitized tumor cells to ABT-737 (Dai and Grant, 2007; Quinn et al., 2011). Additional studies suggest that decreased expression of miRNA-101 results in elevated expression of *Mcl-1* in NSCLC, and consequently poorer prognosis (Su et al., 2009; Wang et al., 2010; Chen et al., 2011; Luo et al., 2012). However, the role of miRNA-101 on drug resistance of lung cancer is not fully understood. Therefore, in the present study, we investigated the effect of miRNA-101 on cellular apoptosis and sensitivity of the A549 NSCLC to ABT-737.

MiRNAs are a kind of non-coding RNAs that play pivotal roles in the regulation of cellular processes such as cell proliferation, differentiation and apoptosis (Yin et al., 2014). Experimental and clinical investigations revealed that up-regulation and down-regulation of miRNAs are linked to the development of malignant phenotype of cancers, including enhancement of cell proliferation, invasion and abrogated apoptosis (Zhang et al., 2017; Wang et al., 2018; Chen et al., 2019). MiRNA-101 is a tumor suppressor miRNA that inhibits the cell proliferation and invasion, induces apoptosis and augments chemosensitivity in several types of cancers including NSCLC (Yin et al., 2014; Han et al., 2018). Here, we demonstrated that single therapy with miRNA-101 obviously reduced the cell growth and survival and increased the extent of apoptosis in A549 cells. Moreover, previous studies have found that miRNA-101 inhibits the cell proliferation and augmented apoptosis in gastric cancer, colon cancer and NSCLC by targeting zinc finger E-box binding homeobox 1 (ZEB1), cyclooxygenase-2 (Cox-2), enhancer of zeste homologue 2 (EZH2) and Mcl-1 (Su et al., 2009; Wang et al., 2010; Luo et al., 2012; Han et al., 2018). However, our findings are in agreement with these reports and further confirm the role of miRNA-101 in the progression of NSCLC as well as the tumor-suppressive effect of this miRNA.

*Mcl-1* is an member of the anti-apoptotic Bcl-2 family proteins that is expressed in various tissues and tumor cells (Schulze-Bergkamen et al., 2006; Sieghart et al., 2006). Mcl-1 involves in apoptosis by blocking cytochrome c-release from mitochondria by sequestering the pro-apoptotic members of the Bcl-2 protein family, e.g. Bid, Bim and NOXA as well as Bak and Bax (Fleischer et al., 2006; Hussain et al., 2007). In apoptotic conditions, specific proteins such as NOXA can displace *Mcl-1* from pro-apoptotic proteins which leading to cytochrome-c release from mitochondria, and subsequently activation of apoptosis (Akgul, 2009; Guoan et al., 2010). Studies have shown that increased levels of Mcl-1 in tumor cells is correlated with high levels of cell survival and development of resistance to diverse chemotherapeutic agents including ABT-737 (Thallinger et al., 2003; Sieghart et al., 2006; Keuling et al., 2009; Quinn et al., 2011). Furthermore, knockdown of *Mcl-1* has been demonstrated to decrease cell survival and reverse drug-resistance of tumor cells (Chen et al., 2007; Chen et al., 2010; Quinn et al., 2011; Lucas et al., 2012). Here, we applied suppression of *Mcl-1* by miRNA-101 to investigate the role of this miRNA in sensitivity of A549 NSCLC cells to the ABT-737. Our findings demonstrated that exposure of the A549 cells to ABT-737 decreased the cell survival rate and induced apoptosis. Transfection of miRNA-101 significantly suppressed the expression levels of Mcl-1 mRNA and synergistically enhanced the cytotoxicity of ABT-737. In addition, miRNA-101 in combination with ABT-737 further enhanced the extent of apoptosis compared to the single therapy. In agreement with our results, Yin et al., (2014) demonstrated that miRNA-101 enhances cisplatin-induced apoptosis via the activation of caspase 3 and reduced colony formation in the A549 cells. Su et al., (2009) reported that miRNA-101 represses the expression of Mcl-1 and sensitizes the liver cancer cells to chemotherapeutic agents. Chen et al., (2011) also demonstrated that ectopic expression of miRNA-101 sensitized NSCLC cell lines to radiation. However, the results of our study are similar to these reports and propose that down-regulated miRNA-101 expression could be related to *Mcl-1* over-expression and ABT-737-resistance of NSCLC cells.

In conclusion, our results provide strong evidence that miRNA-101 could suppress the cell growth and survival and trigger apoptosis in NSCLC through blocking *Mcl-1*. Furthermore, we showed that miRNA-101 enhanced the sensitivity of the lung cancer cells to ABT-737 mediated apoptosis. Therefore, our study suggests that miRNA-101 can be considered as an effective target to reverse ABT-737 resistance in cancer cell.
